# Effects of Chronic Metal Exposure on the Morphology of Beetles Species Representing Different Ecological Niches

**DOI:** 10.1007/s00128-018-02532-7

**Published:** 2019-01-21

**Authors:** Grzegorz Sowa, Tomasz Skalski

**Affiliations:** 1 Institute of Environmental Sciences, Jagiellonian University, Gronostajowa 7, 30-387 Kraków, Poland; 2 Biotechnology Centre, Silesian University of Technology, ul. Krzywoustego 8, 44-100 Gliwice, Poland

**Keywords:** Body size, Metal pollution, Carabidae, Curculionidae, Geotrupidae

## Abstract

To test effects of metal contamination on beetles morphology, specimens from species representing herbivores (*Strophosoma capitatum*), carnivores (*Carabus arcensis* and *C. violaceus*) and detritivores (*Anoplotrupes stercorosus*) were collected from an area polluted with zinc, lead and cadmium and from a control site. Both the length and width of elytra and pronotum were compared. Females of all species from the polluted area were smaller than those from the control site with the average difference of 2.7% (range 0.7%–6%). In contrast, males responded less consistently among species: *A. stercorosus* showed lower size of elytra and pronotum at the polluted area, while in *C. arcensis* only the elytra length and pronotum width were smaller. *C. violaceus* males exhibited smaller elytra length and pronotum length and width at the polluted area. In contrast, no differences between the two sites were found for *S. capitatum* males. Sex differences may originate from different energy investment strategies in females and males related to the reproduction needs. Even if the observed differences in body size were small, the smaller body size in females of all studied species, irrespectively of the guild, is striking and may indicate on lower fitness of a range of species inhabiting metal polluted areas.

Trace metals occur naturally in the Earth’s crust. Some of them are essential for biochemical processes (e.g., copper, iron, zinc), while others are comparatively harmless (gold) or are naturally present in soils at low, non-hazardous concentrations (e.g., cadmium and lead). However, both the essential and non-essential metals can be toxic at high concentrations. The increasing demand of humankind for a range of metals and the associated dynamic industrial development resulted in increased emissions of trace metals into the environment and serious pollution of soils (Ermakov 2004). Chronic exposure to elevated concentrations of such trace metals such as zinc (Zn), lead (Pb), copper (Cu) or cadmium (Cd) may affect fertility and reproduction of organisms (Laskowski and Hopkin 1996), their morphology (Braun et al. 2004) and the life span (Kozłowski and Cichoń 2000). It has been hypothesized that exposure to toxic chemicals, including trace metals at elevated concentrations, causes an increased burden on organism’s energy budget (Sibly and Calow 1989). To prevent toxic or negative effects, internal concentrations of chemicals in organisms must be strictly controlled. Metals cannot be degraded, hence controlling their internal body concentration is restricted to decreased assimilation and/or increased excretion rates while detoxification is based on fixing metal ions in insoluble granules (Marigómez et al. 2002) or metallothioneins (Amiard et al. 2006). Both ways of preventing toxic effects interfere with the energetic budget and may lead to various developmental disorders or ultimately changes in size and structure of populations (Skalski et al. 2010, 2015) due to decreased energy investment in reproduction or survival. Indeed, Maryański et al. (2002) observed that the ground beetles, *Poecilus cupreus*, fed Cd-contaminated housefly larvae in a laboratory experiment showed a decreased body caloric value and size of elytrae, tibiae and rear femora. This has not been, however, confirmed in the field. In fact, Zygmunt et al. (2006) found no effect of metal pollution on caloric body value in the closely related species *Pterostichus oblongopunctatus* in the same metal-polluted area as used in our study. Even more surprisingly, the latter authors noticed a weak yet significant increase in the beetles’ body mass with increasing metal pollution.

Many species are used as bioindicators of pollution and different traits serve as toxicity biomarkers. A bioindicator can be a species or a group of species that react to changes in biotic or abiotic environmental conditions in a way which clearly indicates specific changes. Such species are also good research models for assessing the effects of metals on organisms. In turn, biomarker can be any trait that is sensitive to toxicants in general or to a particular chemical (Kammenga et al. 2000). For instance, morphological changes in ground beetles (Carabidae) were used as the indicator of habitat quality (Lagisz 2008).

The objective of this study was to assess the impact of chronic metal contamination on the morphology of beetle species representing different taxonomic groups and ecological guilds. We hypothesized that beetles inhabiting metal-polluted environments are smaller than those from control population irrespective of species, sex or ecological guild. If current these insights indicate elevated maintenance costs during development indirectly at polluted sites. The tested hypothesis can deliver information about shifts in energy allocation due to toxic stress and, if applicable, sex specific differences. Despite the strong hypothetical background, such shifts are very difficult to measure and experimental proofs supporting the ‘stress hypothesis’ by Sibly and Calow (1989) are almost non-existent. In contrast to some earlier studies, when body mass was used as a proxy of body size, we used the pronotum and elytrae dimensions as more robust measures of long-term effects during larval and pupal stages: body mass and linear dimensions (e.g. elytra length) are often used interchangeably in terms of insects body size. Although these two parameters may be strongly related (mass^0.33^ ≈ length) (Chown and Gaston 2010), this is not always the case. Body mass fluctuates daily and seasonally, depending on actual external (temperature, moisture) and internal (feeding state, pregnancy in females, etc.) conditions, and poorly indicates long-term responses to environmental conditions. On the other hand, the dimensions of the exoskeleton remain permanently fixed in adult insects (Den Nijs and Lock 1996). The differences in soil habitat quality caused by metal pollution should, thus, be reflected in the size of the exoskeleton parts of the adult.

## Materials and Methods

**Fig. 1:**
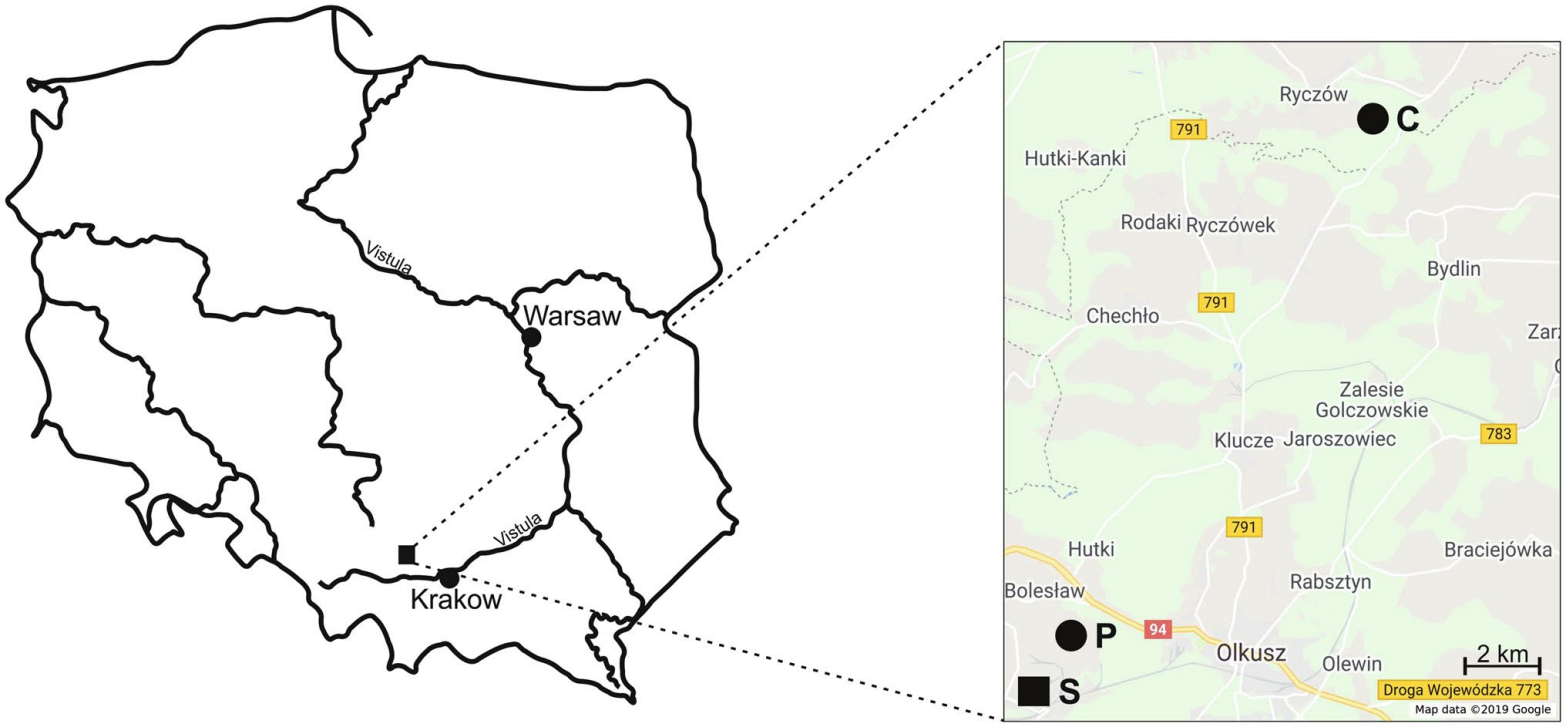
Location of the smelter (S) in Poland and two sampling areas: *P* polluted and *C* control [Google Inc. (2018) https://www.google.com/maps. Accessed 2018]

To account for possible differences in exposure to metals and in energy allocation to detoxification, we decided to study effects of metal pollution on adult beetles representing three different guilds, namely herbivores, carnivores and detritivores. The beetles were collected in high season from May to the end of September (2012) in the vicinity of the ‘Bolesław’ zinc-and-lead smelter near Olkusz city in southern Poland. Two sites were selected for the study: one heavily polluted (P) with metals (mainly Zn, Cd and Pb), located 3.5 km away of the smelter, and the control area (C) 31 km from the smelter (Fig. 1) exhibiting only background concentration of metals (Table 1). The sampling sites were used before in a number of studies on effects of metal contamination on invertebrates and soil microorganism, with the most recent being Azarbad et al. (2013) from which the concentrations of trace metals in soil were taken. Both sampling sites were located in a mixed Scots pine-oak forest (Pino-Quercetum), which is the most common forest formation in Poland (Szafer and Zarzycki 1972). The two sampling areas are 32 km apart and do not differ in altitude – hence the climate and actual weather conditions were the same. Fifty Barber-type traps were placed at each plot in five rows, about 1 m apart from each other (50 m^2^). A detailed description of the study region can be found in Stefanowicz et al. (2014).

**Table 1:** Average concentrations (standard deviations; n = 3) of trace metals in the soil organic layer of the two study sites

Area	Zn (mg/kg)	Cd (mg/kg)	Cu (mg/kg)	Pb (mg/kg)
P	4249 (205)	77.3 (7.80)	41.6 (6.13)	2949 (327)
C	270 (18.6)	3.98 (0.80)	5.17 (0.49)	298 (6.75)
AV	300	4	150	100

*P* polluted and *C* control (taken from Azarbad et al. 2013); *AV* acceptable values for forest soils (Żelichowski 2002)

The beetles were sampled using Barber traps (0.2 L plastic cups, 10 cm diameter, 15 cm deep) filled with the glycol-based engine coolant Borygo (Boryszew ERG, Poland) as a preservative. Collected beetles were transferred to 100 mL containers filled with Line-EtOH – 96% ethanol alcohol, 3% diethyl ether, 1% *tert*-butyl-methyl ether (Linegal Chemicals, Poland) – and transported to the laboratory. Only four species were abundant enough in both study areas to allow for a reliable statistical analysis and these were used in the studies: *Strophosoma capitatum* (Curculionidae, a herbivore), *Carabus arcensis* and *Carabus violaceus* (Carabidae, predators) and *Anoplotrupes stercorosus* (Geotrupidae, a detritivore). The species selected for the study were the most abundant and are considered representative for the studied areas. The total number of individuals used for the analysis was 400. We used 20 specimens per species, sex and site (40 in case of the detritivore). Specimens were placed on 22 cm diameter plastic plates to dry and then photographed using the Nikon SMZ 1500 stereoscopic microscope coupled with Nikon DS-Fi1-U2 camera. Width and length of pronotum and elytrae (PW, PL, EW and EL, respectively) were measured using a biometric kit of the NIS Elements program (Nikon, Japan) to the nearest 0.001 mm. The calibration settings for the magnifications used during measurements were recorded in JPEG 2000 format to recalculate the number of pixels to the length units. In the case of *A. stercorosus* we used specimens collected in 2011 and 2012, while all individuals belonging to the remaining three species were collected in 2012.

To test the beetles for differences in size between populations inhabiting polluted and unpolluted areas we used General Linear Models (GLM) with multivariate analysis of variance (MANOVA). In the first step, we analysed all species and both sexes together, with site, species and sex as independent factors and including their interactions. Because all factors and interactions were highly significant (*p* < 0.0001), in the next step we performed GLM analysis separately for males and females. In males the interaction term was still significant at *p* < 0.003, hence we run separate GLM analysis for each species. We assumed *p* ≤ 0.05 as statistically significant. All statistical analyses were performed using Statgraphics Centurion XVII (StatPoint Technologies, Inc., USA).

## Results and Discussion

**Fig. 2:**
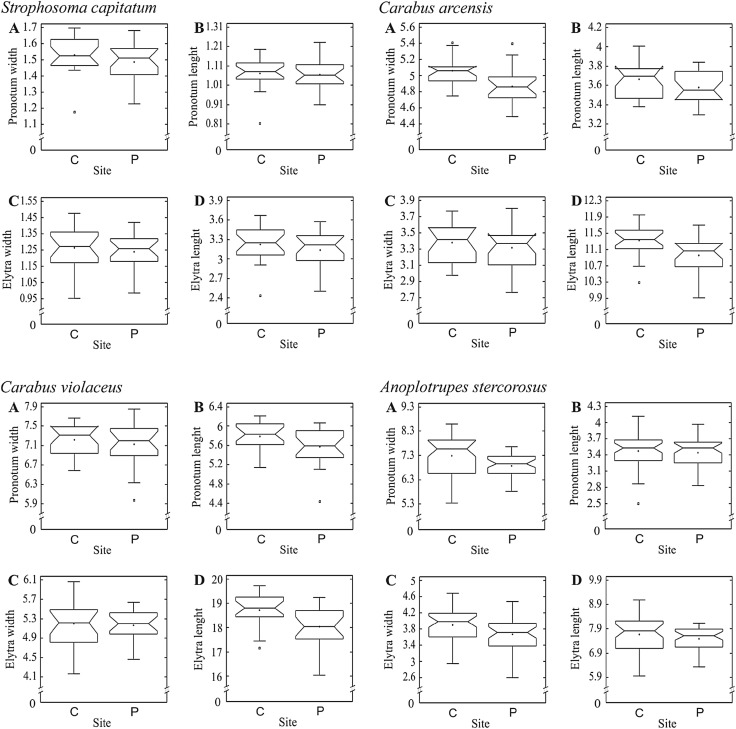
Effects of metal contamination on body size of females of the four beetle species: *Strophosoma capitatum, Carabus arcensis, Carabus violaceus* and *Anoplotrupes stercorosus;***a** – pronotum width, **b** – pronotum length, **c** – elytra width and **d** – elytra length (PW, PL, EW, EL). The graphs indicate median (shorter horizontal line), average (plus sign), second and third quartile (wider horizontal lines), minimum and maximum (whiskers) except for outliers > 1.5 interquartile rage (asterisks); the notch indicates approximate 95% confidence interval for the median. Two study sites: *C* control and *P* polluted

Metal concentrations in soil at the control site were within Polish norms for uncontaminated forest soils for all metals except Pb. Metal concentrations at the polluted site were 8.1 (Cu)–19.4 (Cd) times higher than in control site (Table 1), making the area one of the most contaminated with metals in Poland. Using just four species is certainly a weakness of the study, yet typical for such filed studies, and the clear trends found across the species (see below) should allow for some general conclusions. Metal contamination significantly affected females irrespective of the species (model *p* < 0.0001). The effect was significant for all parameters except EW (*p* = 0.0961) (Table 2). Interactions between the factors were non-significant, indicating that females from all species react in a similar way to metal pollution. Our results have shown that females from the populations inhabiting the contaminated area are generally smaller than those inhabiting the control site (Fig. 2). In females of all species all measures except EW were smaller in populations from the polluted area but the effect was rather small, with an average difference of 2.7% (range 0.7%–6%).

**Table 2:** Results of the general linear models analysis (*p* values) for females from all four species with species and site as independent variable

	Df	PW	PL	EW	EL
Model	7	< 0.0001	< 0.0001	< 0.0001	< 0.0001
Site	1	0.0098	0.0336	0.0961	0.0001
Species	3	< 0.0001	< 0.0001	< 0.0001	< 0.0001
Site × Species	3	0.1512	0.2445	0.3275	0.0770
Residual	192				

*Df* degrees of freedom, *PW* pronotum width, *PL* pronotum length, *EW* elytrae width, *EL* elytrae length

**Fig. 3:**
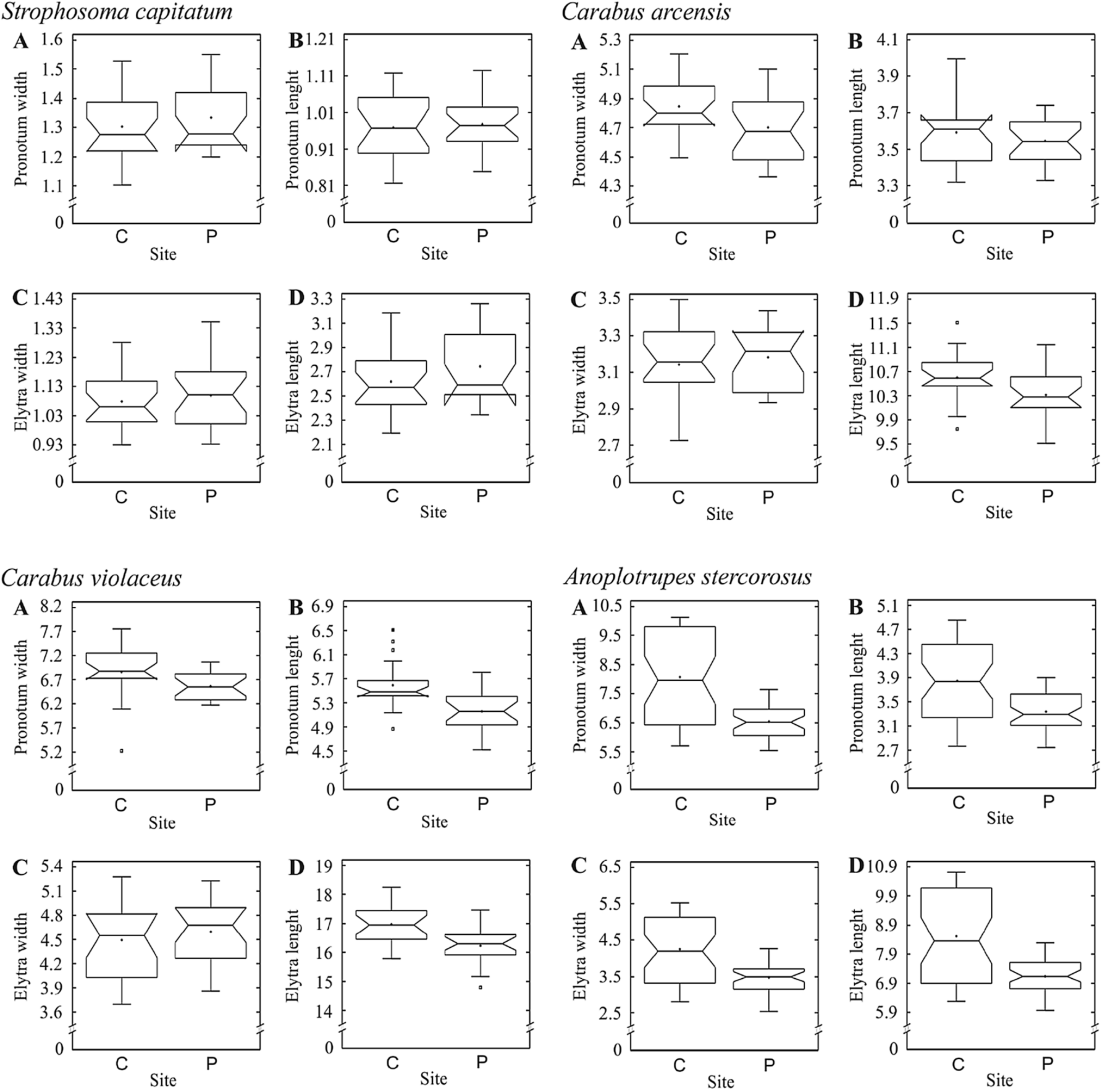
Effects of metal contamination on body size in males of four beetle species: *Strophosoma capitatum, Carabus arcensis, Carabus violaceus* and *Anoplotrupes stercorosus;***a** – pronotum width, **b** – pronotum length, **c** – elytra width and **d** – elytra length (PW, PL, EW, EL). The graphs indicate median (shorter horizontal line), average (plus sign), second and third quartile (wider horizontal lines), minimum and maximum (whiskers) except for outliers > 1.5 interquartile rage (asterisks); the notch indicates approximate 95% confidence interval for the median. Two study sites: *C* control and *P* polluted

Also in case of males the GLM on all species and traits was highly significant (*p* < 0.0001). However, in this case, besides all factors, also the interaction terms were significant (Table 3). The significance of these interactions implies that species react to metal contamination in different ways and, in contrast to females, no general response was observed. Hence, the data were analysed separately for each species (Table 4). These analyses have shown a significant decrease in some parameters in populations inhabiting the polluted area but differing among the species (Fig. 3).

**Table 3:** Results of the general linear models analysis (*p* values) for males from all four species with species and site as independent variable

	Df	PW	PL	EW	EL
Model	7	< 0.0001	< 0.0001	< 0.0001	< 0.0001
Site	1	0.0001	< 0.0001	0.0266	< 0.0001
Species	3	< 0.0001	< 0.0001	< 0.0001	< 0.0001
Site × Species	3	< 0.0001	0.0003	< 0.0001	< 0.0001
Residual	192				

*Df* degrees of freedom, *PW* pronotum width, *PL* pronotum length, *EW* elytrae width, *EL* elytrae length

**Table 4:** Results of the general linear models analysis (*p* values) for males with each species being analysed separately, site served as independent variable

	PW	PL	EW	EL
*Strophosoma capitatum*	0.3790	0.7238	0.5674	0.1149
*Carabus arcensis*	0.0425	0.3583	0.5360	0.0208
*Carabus violaceus*	0.0351	0.0005	0.4592	0.0010
*Anoplotrupes stercorosus*	< 0.0001	< 0.0001	< 0.0001	< 0.0001

*PW* pronotum width, *PL* pronotum length, *EW* elytrae width, *EL* elytrae length

Changes in environmental conditions are frequently connected with shifts in energy budget of organisms, what is often reflected in changes of body size. In their classic work, Sibly and Calow (1989) showed that any stress, including those caused by pollution, shifts the trade-off curve linking growth rate with mortality rate, which influences the optimal strategy for energy allocation. Depending on physiological limitations and specific environmental pressures, this can mean a shift towards higher mortality, lower productivity or the combination of both. Decreased availability of resources for maintenance can be also reflected in worsened health status or morphological changes of the organism (Braun et al. 2004; Kozłowski and Cichoń 2000). In heavily contaminated environments organisms likely increase the share of energy resources spent on detoxification processes. Thanks to these processes, living in a contaminated area does not have to be lethal (Grześ 2009; Dunger 1989) but the shift in energy allocation must be reflected in some individual traits. Our results have shown that females of all studied species inhabiting the metal polluted area had smaller sizes of both elytra and pronotum (Fig. 2). Although the differences between individuals from control and polluted areas were small, the effect was statistically significant, indicating on high statistical power of the study and the consistency of results, especially considering the limited sample sizes. In fact, one should not expect larger effects for at least two reasons: (1) body size is a strong determinant of fitness and, as such, is protected by evolutionary pressures and (2) diverting energy from growth to detoxification most probably means well below 5% of the total energy budget as overall protein turnover is estimated at ca. 5% of the metabolic rate. Nevertheless, even minor differences in body size are usually reflected in fitness, indicating on lower fitness of the beetles inhabiting the contaminated area.

As the main difference between the two habitats was the extreme difference in pollution with Zn, Cd, and Pb, we may assume that the smaller body size in females resulted from the allocation of energetic resources from growth to maintenance (Kozłowski 1991, Sibly and Calow 1989). Although other causes for this difference between the populations cannot be excluded because the results are based on comparing the four species originating from only two different communities, the similarity of the studied habitats in terms of soils, forest types and climate is a strong indication that the pollution was an important, if not the sole, factor behind the observed differences. Also the fact that differences in size of body parts were similar in females representing such diverse species as carabids, weevils and scarabaeids strengthens this hypothesis. This is also in line with the studies by Maryański et al. (2002) and Lagisz (2008) who found that in laboratory studies the size of elytra decreased with increasing metal intoxication.

The reaction of females was apparently more uniform than in males. This does not seem surprising considering the different pressures on growth and survivorship in the two sexes. While males can die soon after fertilizing a female, females have to survive until the eggs are fully developed and ready to be laid in order to pass the genes to subsequent generations (Katsuki et al. 2012). The possibility to achieve reproductive success by males even after relatively short life may mean less uniform pressures on shifts in energy budgets of individuals and species. Indeed, in case of males, each species reacted differently to the pollution (Fig. 3). While *A. stercorosus* had all four measures of the body size smaller in the polluted area, in *C. violaceus* the length of elytra and the length and width of pronotum were reduced, and in *C. arcensis* only length of elytra and width of pronotum were smaller. Both latter species are predators and the only element of their life history strategies that differentiates them is the reproductive strategy: *C. arcensis* is a spring breeder, while the *C. violaceus* reproduces in autumn. The similar effect of the contamination in males of both species may indicate that this difference in species biology does not play an important role in shaping the energetic trade-offs in carabids. In contrast, in *S. capitatum* no difference was found between the beetles inhabiting the contaminated and control sites. Perhaps, *S. capitatum*, as a herbivore, is less exposed to metal contamination than carnivorous or detritivorous species.

These results suggest that due to the lower investment of energy to reproduction in males than in females and no selection pressure to survive beyond female fertilization (Simmons 2001), males have broader range of possible locally optimal strategies in polluted environments. For example, the lack of difference in size of the major body parts between males of *S. capitatum* from the two habitats may suggest that in this species it is the male size rather than longevity what determines its reproductive success (Simmons et al. 1999; Kotiaho 2002). If this is the case, indeed the expected optimal strategy in polluted environment should be to grow fast, reach as large body size as possible, fertilize as many females as possible and die (Roff 1992, 2000). This strategy is realized by not investing in detoxification and allocating all available resources to growth.

We believe that the study, although of a limited scope due to comparing only two areas representing two extremely different contamination levels, has provided interesting results and generated important hypotheses to be tested in further studies. The study showed that chronic exposure to high concentrations of trace metals may reduce body size in beetles representing different species and life strategies and that the effect is more consistent in females than in males. We hypothesize that the observed trend results from shifts in energy allocation due to metal detoxification and that the observed differences between males and females stem from different priorities in energy investment strategies between the sexes. Even if the observed effects are small in size, they may affect beetles fitness.
